# The age of onset and evolution of Braak tangle stage and Thal amyloid pathology of Alzheimer’s disease in individuals with Down syndrome

**DOI:** 10.1186/s40478-018-0559-4

**Published:** 2018-07-04

**Authors:** Yvonne S. Davidson, Andrew Robinson, Vee P. Prasher, David M. A. Mann

**Affiliations:** 1 Institute of Brain, Behaviour and Mental Health, Faculty of Medical and Human Sciences University of Manchester, Salford Royal Hospital, Stott Lane, Salford, M6 8HD England; 2 South Birmingham Community NHS Trust, Birmingham, UK; 3 Liverpool John Moores University, Liverpool, UK

**Keywords:** Alzheimer’s disease, Down syndrome, Amyloid, Tau

## Abstract

While post mortem studies have identified the major cell types and functional systems affected in Alzheimer’s disease (AD) the initial sites and molecular characteristics of pathology are still unclear. Because individuals with Down syndrome (DS) (trisomy 21) develop the full pathological changes of AD in a predictable way by the time they reach middle to late age, a study of the brains of such persons at different ages makes an ideal ‘model system’ in which the sites of earliest onset of pathology can be detected and the subsequent progression of changes be monitored. In the present study we have examined the brains of 56 individuals with DS ranging from new-born to 76 years for the presence of amyloid and tau pathology in key cortical and subcortical regions. Amyloid pathology was found to commence in the late teens to twenties as a deposition of diffuse plaques initially within the temporal neocortex, quickly involving other neocortical regions but only reaching subcortical regions and cerebellum by the late forties. Cerebral amyloid angiopathy did not regularly commence until after 45–50 years of age. Tau pathology usually commenced after 35 years of age, initially involving not only entorhinal areas and hippocampus but also subcortical regions such as locus caeruleus (LC) and dorsal raphe nucleus (DRN). Later, tau pathology spread throughout the neocortex reaching occipital lobes in most instances by mid-50 years of age. Such a pattern of spread is consistent with that seen in typical AD. We found no evidence that tau pathology might commence within the brain in DS before amyloid deposition had occurred, but there was limited data that suggests tau pathology in LC or DRN might predate that in entorhinal areas and hippocampus or at least be coincident.

## Introduction

Alzheimer’s disease (AD) is characterised pathologically by the abundant presence of deposits of amyloid β protein (Aβ) in the brain parenchyma in the form of amyloid plaques, and in blood vessel walls as cerebral amyloid angiopathy (CAA), and neurofibrillary tangles (NFT) composed of hyperphosphorylated tau proteins [19, 35]. While post mortem studies based on established cases of AD have identified the major cell types and functional systems affected by the disorder, the initial sites of pathology are still unclear. According to the original and modified Braak stageing protocols [4, 7], the earliest sites of tau pathology lie within the entorhinal and transentorhinal cortex (stage I), spreading to hippocampus (stage II), temporal cortex (stage III) and eventually to other regions of cerebral cortex (stage IV), finally reaching visual association cortex (stage V) and primary visual cortex (stage VI). Conversely, the order of amyloid plaque deposition [46] commences within neocortical regions, particularly within the temporal lobe (phase 1), and spreads to involve allocortical regions such as the hippocampus and amygdala (phase 2), then into subcortical regions (phase 3), brain stem (phase 4) and finally cerebellum (phase 5).

However, this concept of the primacy and spread of tau pathology in AD has recently been questioned. From a study of 42 cognitively normal individuals under 30 years of age, Braak and Del Tredici suggested that the earliest site of tau pathology in AD might actually occur within certain subcortical nuclei, notably the locus caeruleus (LC), predating that within the entorhinal area [6]. Of these 42 individuals, deposits of Aβ were present in only 1 person, a 17-year old with Down Syndrome (DS), simultaneously challenging the concept of the amyloid cascade hypothesis of AD [14]. Another study by this same research group of 2332 unselected post mortems ranging from 1 to 100 years of age [8] found tau pathology in 58 cases, in the absence of Aβ deposition in all but one instance (again the same case of DS as included in [6]). Tau pathology was essentially restricted to those individuals aged 30 years or less, and occurred in LC and certain other non-cortical nuclei such as the upper raphe in the brainstem and the nucleus basalis of Meynert in the basal forebrain, without there being any entorhinal/cortical involvement. These data were interpreted as providing evidence for there being stages in the pathological process of AD that predate those affecting entorhinal/transentorhinal areas, and that changes in these subcortical nuclei may represent the very earliest sites of damage to the brain in AD. Nevertheless, from these kinds of observational studies it is not possible to predict whether any of the affected individuals would have gone on to develop AD had they survived longer. Moreover, the lack of an (inducible) animal model that fully recapitulates the pathological process of AD precludes experimental studies of the time course of the development of AD pathology.

On the other hand, it has long been known that individuals with Down syndrome (DS) associated with an additional copy of chromosome 21 (trisomy 21) develop the full pathological changes of AD by the time they reach middle age, and sometimes as young as 40 years [9, 13, 16, 18, 28–30, 38, 42, 50]. Hence, a study of the brains of such persons at different ages makes an ideal ‘model system’ in which the earliest sites of Alzheimer-type pathology can be observed and the subsequent progression of such changes be monitored. As with individuals with duplications in *APP*, DS is associated with an overexpression of APP protein, and therefore it might be argued that DS represents a model of Aβ hyperproduction rather than AD per se. Nonetheless, it still remains closer in pathological terms to AD than any other human condition or experimental paradigm. In the present study we have examined the brains of 56 individuals with DS ranging from new-born to 76 years for the presence of amyloid and tau pathology in certain cortical and subcortical regions and have charted the onset and progression of these, and other, pathologies in each brain region with age, and in relationship to each other.

## Material and methods

Serial wax sections were cut at 6 μm thickness from formalin fixed tissue blocks from the brains of 56 individuals with DS (34 males, 22 females) ranging from new-born to 76 years (mean 48.0 ± 20.2 years). There was no significant difference in mean age between males and females (males, 48.3 ± 20.7 years; females, 47.4 ± 19.8 years). Thirteen of the 56 cases were drawn from the Manchester Brain Bank (MBB), 7 were obtained from Institute of Psychiatry (IOP) Brain Bank, London, 22 from the Thomas Willis Brain Bank (TWBB), Oxford, with the remaining 14 being obtained through Professor V P Prasher at University of Birmingham. All brains had been obtained at autopsy through appropriate consenting procedures with Local Ethical Committee approval. Selected case details are provided in Table 1. Details concerning karyotyping were only available for 13 individuals, but all 13 were a full trisomy 21 (Table 1).

**Table 1:** Selected demographic, genetic and neuropathological details for 56 individuals with Down syndrome

Case ID#	UKBBN ID#	gender	age	karyotype	*APOE*	Thal	Braak	Allen CAA	Thal CAA
1	na	M	0.01	na	na	0	0	0	0
2	na	F	0.1	na	na	0	0	0	0
3	na	M	1.6	na	na	0	0	0	0
4	na	M	1.6	na	na	0	0	0	0
5	na	F	3	na	na	0	0	0	0
6	na	M	11	na	na	0	0	0	0
7	na	F	13	na	na	0	0	0	0
8	BBN_2966	M	13	47XY21	3,3	1	0	0	0
9	na	M	23	na	na	0	0	0	0
10	na	F	27	na	na	1	0	0	0
11	na	M	35	na	na	2	0	0	0
12	na	M	36	na	na	5	II	0	0
13	BBN_2963	F	37	47XX21	3,4	5	I	0	0
14	na	F	39	na	na	2	b*	0	0
15	na	M	42	na	na	5	V	1	1
16	BBN_17186	F	47	na	na	5	VI	3	2
17	BBN_17189	M	47	na	na	5	III	1	1
18	BBN_17178	F	49	na	3,4	4	III	1	1
19	BBN_2981	M	50	47XY21	3,4	3	II	1	1
20	na	F	50	na	na	0	b*	0	0
21	na	M	51	na	na	5	VI	1	1
22	BBN_2968	M	53	47XY21	3,3	5	III	1	1
23	BBN_3356	F	53	na	3,4	5	VI	2	1
24	BBN_16954	M	54	na	2,4	5	VI	3	2
25	BBN_3365	M	55	na	3,4	5	VI	2	1
26	na	M	55	na	na	5	V	1	1
27	BBN_3438	F	56	na	3,3	5	VI	1	1
28	BBN_16273	F	56	na	3,4	5	VI	1	1
29	BBN_2978	M	57	47XY21	3,3	5	V	3	2
30	na	M	57	na	na	5	VI	1	1
31	BBN_3020	F	58	47XX21	3,3	5	V	1	1
32	BBN_3355	M	58	na	3,4	5	VI	3	2
33	BBN_3358	F	58	na	3,3	5	VI	2	1
34	BBN_3437	F	58	na	2,3	5	V	1	1
35	BBN_3353	M	59	na	2,3	5	VI	3	2
36	BBN_3363	M	59	na	3,3	5	VI	2	1
37	BBN_2990	M	60	47XY21	3,3	5	VI	2	1
38	BBN_3364	M	60	na	3,3	5	VI	2	1
39	na	F	60	na	na	5	0	0	0
40	na	M	60	na	na	5	VI	2	1
41	BBN_16835	M	60	na	3,3	5	III	2	1
42	BBN_3352	F	61	na	2,3	5	VI	3	2
43	BBN_3439	F	61	na	3,4	5	VI	2	1
44	BBN_2964	F	62	47XX21	3,3	5	V	3	2
45	BBN_2965	M	62	47XY21	3,3	5	V	1	1
46	BBN_2967	F	62	47XX21	3,3	5	IV	3	2
47	BBN_3354	M	62	na	3,3	5	VI	3	2
48	BBN_3441	M	62	na	2,3	5	VI	3	2
49	na	M	63	na	na	5	VI	1	1
50	BBN_2969	M	64	47XY21	3,3	5	IV	1	1
51	BBN_3440	F	64	na	3,3	5	VI	2	1
52	BBN_2975	M	65	47XY21	3,3	5	V	1	1
53	BBN_17004	M	65	na	3,3	5	V	1	1
54	na	M	66	na	na	5	V	1	1
55	na	F	69	na	na	5	VI	1	1
56	BBN_2985	M	76	47XY21	2,3	5	III	3	2

Thal = Thal phase of amyloid deposition and Braak = Braak tau stage. CAA phenotype was assessed in accordance with Allen et al. (2014) and Thal et al. (2010). UKBBN = UK Brain Bank Network ID number, na = not available/applicable

Brain regions investigated were frontal cortex (BA8/9), temporal cortex (BA 21/22) to include the entorhinal cortex and hippocampus (at the level of the geniculate bodies), occipital cortex (BA17/18), cerebellar cortex, and brainstem to include substantia nigra (SN), LC and DRN (where available) and corpus striatum (CS) (caudate nucleus and putamen). Adjacent sections were routinely stained by haematoxylin and eosin, and immunostained for tau (using mouse monoclonal antibody, AT8 (Innogenetics, Antwerp, Belgium, 1:750)) and Aβ (using mouse monoclonal antibody, 4G8 (Covance Research Products Inc., Dedham MA, USA, 1:3000). Following 4G8 and AT8 immunostaining, all cases were staged as to Thal phase of Aβ deposition [46] and Braak stage of neurofibrillary degeneration using the revised Braak criteria for AT8 immunostained paraffin sections [7]. Further sets of sections of brainstem (substantia nigra (*n* = 27) and/or locus caeruleu (*n* = 56)) and temporal lobe with entorhinal cortex and hippocampus were immunostained for phosphorylated α-synuclein using a polyclonal anti-psyn1175 antibody [36] at 1:1000 dilution (kind gift of Dr. Masato Hasegawa at Tokyo Metropolitan Institute of Medical Science, Japan), and an additional set of sections of temporal cortex with hippocampus was immunostained for phosphorylated and non-phosphorylated TDP-43 using a polyclonal antibody (10782–2-AP Proteintech, Manchester, 1:1000). Those sections immunostained for tau, α-synuclein and TDP-43 underwent antigen retrieval by pressure cooking in citrate buffer for 30 min, reaching 120 degrees Celsius and > 15 kPa pressure whereas sections immunostained for Aβ were subject to incubation in 90% formic acid for 5 min as pretreatment.

Sections were also examined microscopically for the appearance, severity and topographical distribution of immunostaining of Aβ within brain parenchyma (as amyloid plaques) and cerebral vessels (as CAA). A five-point scoring system [31, 37] was employed to separately grade the severity of plaques and CAA.

### Plaques

- Grade 0 - no amyloid plaques in present.
- Grade 1 - A few amyloid plaques in each low power (× 10 microscope objective) field.
- Grade 2 - A moderate number of amyloid plaques in each low power field.
- Grade 3 - Many dispersed amyloid plaques in each low power field.
- Grade 4 - Very many, densely packed amyloid plaques in each low power field.

### CAA

- Grade 0 - No CAA in blood vessel walls in leptomeninges or brain parenchyma.
- Grade 1 - Occasional blood vessels with CAA in leptomeninges and/or brain parenchyma, usually not occupying the full thickness of the wall.
- Grade 2 - A moderate number of blood vessels with CAA in leptomeninges and/or brain parenchyma, some occupying the full thickness of the wall.
- Grade 3 - Many blood vessels with CAA in leptomeninges and/or brain parenchyma, some occupying the full thickness of the wall.
- Grade 4 - Most or all blood vessels with severe CAA in leptomeninges and/or brain parenchyma, occupying the full thickness of the wall.

CAA subtype, based on examination of frontal, temporal and occipital cortex in sections immunostained for Aβ, was assigned to all cases. Two classification systems were employed, as previously described [2, 47]. According to Allen et al. [2], Type 1 describes cases predominantly with many diffuse and cored Aβ plaques, throughout the cerebral cortex, in which CAA is confined within leptomeningeal vessels. Type 2 describes cases where, along with many diffuse and cored Aβ plaques, CAA is present in both leptomeningeal and deeper penetrating arteries, especially within occipital cortex. Type 3 describes cases where capillary CAA is present along with arterial CAA, especially within primary visual cortex, but with relatively few Aβ plaques. Type 4 describes a predominantly vascular phenotype, where Aβ deposition is much more prevalent in and around blood vessels throughout the brain and Aβ plaques are scarce or absent. According to Thal et al. [47] type 1 CAA includes both leptomeningeal and parenchymal vascular involvement and type 2 additionally involves capillaries.

The severity of overall tau (AT8) pathology (which included neurofibrillary tangles, neuritic plaques and neuropil threads) was assessed as:

- Grade 0 - no tau pathology present.
- Grade 1 - few, widely scattered neurofibrillary tangles and/or neuropil threads in each low power (× 10 microscope objective) field.
- Grade 2 - a moderate number of clustered neurofibrillary tangles and/or neuropil threads in each low power field.
- Grade 3 - many clustered neurofibrillary tangles and/or neuropil threads in each low power field.
- Grade 4 - many densely packed neurofibrillary tangles and/or neuropil threads in each low power field.

A similar system was employed to score α-synuclein immunostained Lewy body and Lewy neurite pathology in SN and LC, entorhinal and temporal cortex, and for TDP-43 immunopositive neuronal cytoplasmic inclusions in the dentate gyrus of the hippocampus and fusiform gyrus of the temporal lobe.

### APOE genotyping

DNA was extracted from frozen cerebellum (or frontal cortex when cerebellum was not available) by routine methods; frozen tissues were not available for genotyping in 24 cases (Table 1). *APOE* genotyping was performed on all samples using method of Wenham et al. [49].

### Statistical analysis

All data analysis was performed using SPSS v 21.0. Levels of significance were two-tailed and set at *p* < 0.05.

## Results

### Histological changes

#### Amyloid pathology

**Fig. 1:**
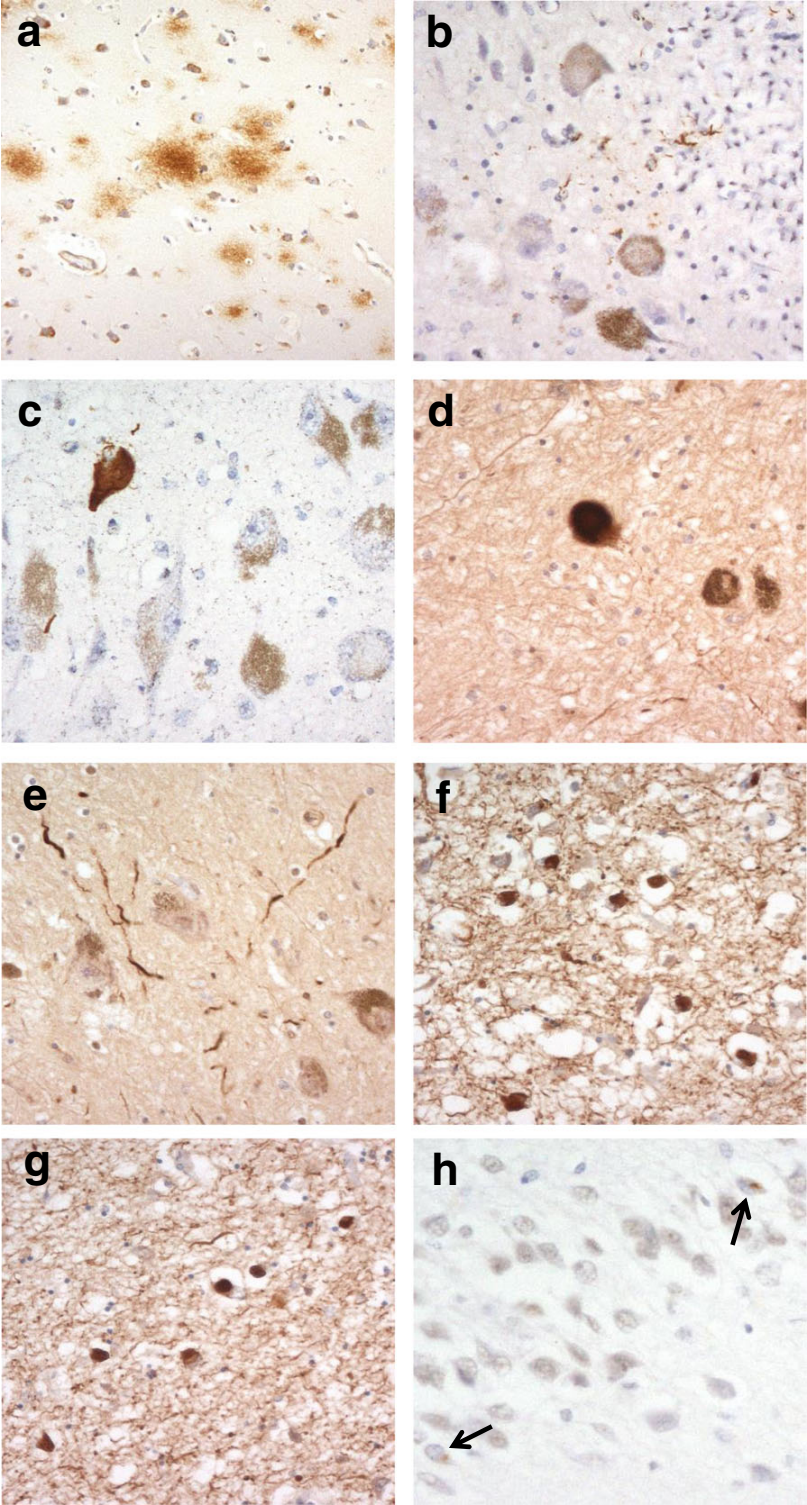
Pathological changes in Down syndrome. In case #8, aged 13 years, amyloid deposits are present in the temporal cortex as diffuse plaques in the absence of any tau pathology (**a**). A few tau positive neurites are present in locus caeruleus in case #14 (**b**) and a single tau positive neurofibrillary tangle is seen, again in locus caeruleus, in case #20 (**c**), in the absence of any tau pathology elsewhere in the brain. A few α-synuclein positive Lewy bodies (**d**) and Lewy neurites (**e**) are present in the substantia nigra in case #45, aged 62 years, but these are more densely present in entorhinal cortex (**f**) and temporal neocortex (**g**) of the same case. Sparse TDP-43 neuronal cytoplasmic inclusions (arrowed) are seen in dentate gyrus granule cells in case #43, aged 61 years (**h**). Immunoperoxidase-haematoxylin; × 250 microscope magnification (**a**) × 400 microscope magnification (**b**–**h**)

**Fig. 2:**
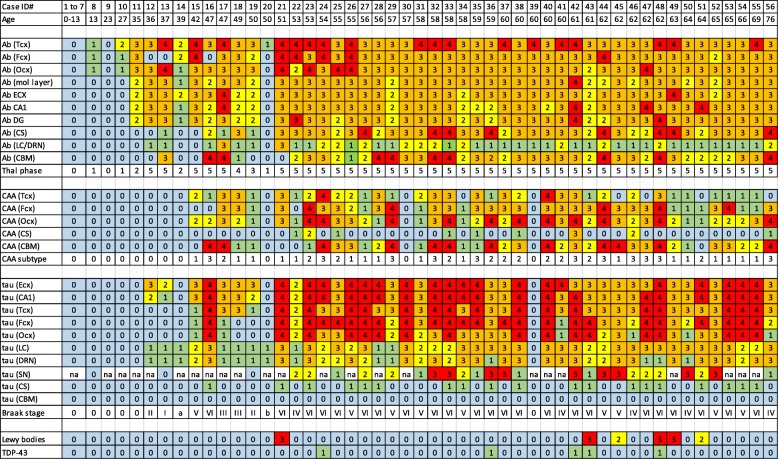
‘Heat map’ illustrating the onset and progression of amyloid plaque, CAA and tau pathology across the different brain regions for the 56 cases of Down syndrome. Box colours indicate increasing severity of pathological change from blue through to red. Numbers in boxes are derived from scoring systems described in the text. Tcx = temporal cortex, Fcx = frontal cortex, Ocx = occipital cortex, Ecx = entorhinal cortex, h = molecular layer of hippocampus, CA1 = CA1 region of hippocampus, DG = dentate gyrus of hippocampus, CS = corpus striatum, LC = locus coeruleus, DRN = dorsal raphe nucleus, SN = substantia nigra, CBM = cerebellum, Ab = amyloid deposits (plaques), CAA = cerebral amyloid angiopathy, tau = tau tangles

Of the 56 individuals, 8 (cases #1–7 and 9) showed no amyloid plaques whatsoever in any brain region examined. Five of these (cases #1–5) were aged 3 years or under, two (cases #6 and 7) were aged 11 and 13 years, and one (case #9) was 23 years of age. Of the remaining 48 individuals, three, aged 13, 27, and 50 years (cases #8, 10 and 20, respectively), showed only rare to moderate numbers of diffuse amyloid plaques (Fig. 1a). In the 27 and 50-year olds these occurred within the temporal cortex alone, but were also present in frontal and occipital cortex in the 13-year old. None were present in the hippocampus, corpus striatum, cerebellum or brainstem of any of these 3 individuals. The other 45 individuals all showed either a moderate number of, or many, amyloid plaques (Fig. 2). However, in 5 of these (cases #11–14 and 19, aged 35, 36, 37, 39 and 50 years, respectively) plaques were mostly diffuse with only few cored, neuritic amyloid plaques being seen within the temporal neocortex and hippocampus, whereas in all other individuals over 50 years of age plaque density and morphology was similar to that typically seen in AD. Hence, cases #1–7 and #9 (where no amyloid plaques were present) corresponded to Thal phase 0, cases #8, 10 and 20 to Thal phase 1, cases #11 and 14 to Thal phase 2, case #19 to Thal phase 3, case #18 to Thal phase 4 with all other cases corresponding to Thal phase 5 (Fig. 2).

#### Cerebral amyloid angiopathy

Fifteen (of twenty) individuals aged 50 years or below showed no CAA at all. CAA was seen only in 5 individuals (cases #15–19) (Fig. 2). In case #15 (aged 42 years) CAA sparsely affected leptomeningeal arteries in the temporal and occipital cortex, but CAA was also present in a few leptomeningeal arteries of the frontal, temporal and occipital cortex and cerebellum in case #19. In the other 3 individuals (cases #16–18, aged 47, 47 and 49 years) CAA was moderate to severe and was present in leptomeningeal arteries in the frontal (except case #16), temporal and occipital cortex and cerebellum (all 3 cases). Cases #15, 18 and 19 therefore displayed type 1 CAA [2]. In case #17 (aged 47 years) CAA also involved parenchymal arteries (type 2) and parenchymal arteries and capillaries (type 3 CAA) in case #16.

All 36 individuals over 50 years of age (except cases #30 and 39, aged 57 and 60 years) showed some degree of CAA (Fig. 2). In 14 individuals this was confined to leptomeningeal arteries (CAA type 1), being only mild to moderate in 10 of these, but severe or very severe in the other 6. In 10 individuals, CAA involved intraparenchymal arteries as well as leptomeningeal arteries (CAA type 2), at least in occipital cortex, often also in frontal and/or temporal cortex, but not in cerebellum. In the remaining 10 individuals there was capillary involvement as well as leptomeningeal and parenchymal artery involvement, again always in the occipital cortex, but occasionally also in the frontal cortex.

Overall, therefore, CAA was present in 39 individuals. According to Allen et a criterial [2] this was present as type 1 CAA in 17 of these (44%), type 2 in 11 individuals (28%) and type 3 in 11 individuals (28%) (Table 1, Fig. 2). According to Thal et al. criteria [47], CAA was present as type 1 in 72% individuals and type 2 in 28% individuals (Table 1).

Mild CAA was seen in small arteries in CS in only 9 individuals (Fig. 2).

#### Tau pathology

Of the 56 individuals, 14 showed no tau tangles or neuropil threads whatsoever in entorhinal cortex, hippocampus or neocortex. Eleven of these (cases #1–11) were aged 35 years or under, one (case #14) was aged 39 years, one (case #20) was 50 years of age and one (case #39 was 60 years of age. Interestingly, in cases #14 and #20, there was scant tau neuritic (Fig. 1b) or neurofibrillary (Fig. 1c) pathology in LC, but without any involvement of cortical or other subcortical structures. Such cases might be classed as pretangle/prodromal stage ‘a’ or ‘b’, respectively (see [6, 8] – a stage recently postulated to predate Stage I in earlier stageing systems [4, 7]. In case #39 no tau pathology at all was seen in any brain region.

Of the other 42 individuals, 10 (cases # 12, 13, 17–19, 22, 41, 46, 50 and 56 aged 36, 37, 47, 47, 50, 53, 60, 62, 64 and 76 years, respectively) showed only a moderate number of, or many tangles within the hippocampus (and entorhinal cortex), with only rare, or a moderate number of, tangles in the temporal, frontal or occipital cortex. These were considered to be at Braak stages II-IV. The remaining 32 individuals (30 of whom were over 50 years of age) showed a moderate number of, many, or very many, tangles in all neocortical regions and hippocampus, similar in appearance, distribution and degree to that typically seen in AD, and were assessed as being at Braak stages V or VI (Fig. 2).

Tau pathology was also investigated in SN in 27 cases where this region was available. No tau pathology was present in any case under 50 years of age, but rapidly developed thereafter such that this was present as neurofibrillary tangles and neuropil threads in all cases examined who were older than 50 years of age, ranging from moderate numbers of both through to there being very many present (Fig. 2).

No tau pathologies consistent with Ageing Related Tau Astrogliopathy (ARTAG) [22] or Argyrophilic Grain Disease [5] were seen in any of the studied cases.

#### α-Synuclein pathology

a few to a moderate number of α-synuclein immunopositive Lewy bodies (Fig. 1d) and Lewy neurites (Fig. 1e) were present in SN and/or LC in 5 cases (#21, 43, 45, 48 and 49), all over 50 years of age, but both pathologies were numerous in the entorhinal cortex (Fig. 1f) and moderately present in the temporal cortex (Fig. 1g), in the same 5 cases and also in case #51 where none were present in the SN or LC. Loss of neurones from SN was generally absent or sparse, even in those cases where Lewy body pathology was present.

#### TDP-43 pathology

TDP-43 pathology was absent from hippocampus and fusiform gyrus/temporal cortex in all but 5 cases (cases #24, 36, 42, 43 and 48, aged 54, 59, 61, 61 and 62 years, respectively). In these latter 5 cases sparse granular neuronal cytoplasmic inclusions were present in cells of the dentate gyrus of the hippocampus (Fig. 1h), but none were seen in fusiform gyrus or temporal cortex. In 3 cases (cases #42, 43 and 47) hippocampal sclerosis of CA1 was also present.

#### Other pathologies

We carefully examined routine haematoxylin eosin stained sections for evidence of small vessel disease, microinfarctions, overt microbleeds or intracerebral haemorrhage but found none to be consistently present in any patient or CAA subtype.

#### Early sites of pathological changes

Figure 2 shows a ‘heat map’ illustrating the earliest sites, and progression, of amyloid plaque, CAA and tau pathology across the different brain regions for the 56 cases.

Amyloid pathology commences in late teens to late twenties as a deposition of diffuse plaques initially within temporal lobe (Thal phase 1), quickly involving other neocortical regions and hippocampus (Thal phase 2), but only reaching subcortical regions (Thal phases 3 and 4) and cerebellum (Thal phase 5) by the late forties. After 50 years of age, every region of the brain, including cerebellum and corpus striatum, is moderately or heavily invested with amyloid plaques (Thal phase 5). Interestingly, CAA does not regularly commence until after 45–50 years of age. In terms of tau pathology and Braak stageing, there is a progressive ‘spread’ of neurofibrillary pathology throughout the brain, usually commencing after the age of 35 years. This initially involves not only entorhinal areas and hippocampus but also subcortical regions such as LC and DRN, sometimes in the absence of entorhinal involvement. Later, tau pathology spreads throughout the neocortex reaching occipital lobes (Braak stages V or VI) in most instances by mid-50 years of age. Lewy body pathology and TDP-43 pathological changes were infrequently present and generally only seen in individuals over 50–60 years of age.

#### APOE genotyping

*APOE* genotypes are given in Table 1. There were 5 individuals bearing ε2ε3 genotype, 18 bearing ε3ε3 genotype, 8 bearing ε3ε4 genotype and 1 bearing ε2ε4 genotype. There were no ε2 or ε4 homozygotes. These genotypes provided *APOE* ε2 and ε4 allele frequencies of 7.8 and 14.0%, respectively. However, age at death was earlier in those individuals bearing one *APOE* ε4 allele (52.6 ± 6.9 years) compared to those bearing only *APOE* ε3 alleles (57.8 ± 11.6 years, *p* = 0.158) and those with *APOE* ε2 alleles (63.2 ± 7.3 years, *p* = 0.019), the latter, in turn, being later than in those individuals bearing only *APOE* ε3 alleles (*p* = 0.232). There were no associations between *APOE* ε2 or ε4 alleles and the severity of Aβ plaque deposition (Thal phase), CAA subtype or severity of tau pathology (Braak stage).

## Discussion

Although post mortem studies of established cases of AD have identified the major cell types and functional systems affected by the disorder, the initial sites of pathology are still unclear. According to the 2006 Braak stageing protocol [7], the earliest sites of tau pathology lie within the entorhinal and transentorhinal cortex (stage I), spreading to hippocampus (stage II), temporal cortex (stage III) and eventually to other regions of cerebral cortex (stage IV), finally reaching visual association cortex (stage V) and primary visual cortex (stage VI). However, this classification has recently been modified and stages prior to the entorhinal involvement affecting subcortical nuclei such as LC and DRN (pre-tangle/prodromalstages a-c) in isolation from any cortical involvement have been proposed [6, 8]. Conversely, the order of amyloid plaque deposition [46] commences within neocortical regions, particularly within the temporal lobe (phase 1), and spreads to involve allocortical regions such as the hippocampus and amygdala (phase 2), then into subcortical regions (phase 3), brain stem (phase 4) and finally cerebellum (phase 5). However, even though both of these protocols were based on a reasonably large number of demented and non-demented individuals (83 individuals were included in the original Braak tau stageing in 1991, and again in 2006 in the modified criteria, with 47 being employed for Thal phase protocols) the data generated still represents an assimilation of single time point, cross sectional observations into a continuum of change. The stageing protocols are therefore based on the presumption that ‘normal’ cases with limited, and presumed early, non-clinical pathological changes would have progressed into ones with dementia and fully developed pathology had they lived longer, and conversely those with full blown AD pathology would have flowed through the same, more limited, stages earlier in the development of disease. While this is a reasonable presumption, it still remains as such given the fact that the data are generated from a heterogeneous collection of heathy and demented individuals where the same risk factors for disease onset and progression may not necessarily apply. The advantage of studying individuals with DS lies mainly in the fact that they are a more homogenous study group who largely share the same genetic (and to some extent environmental) risk of disease, and are therefore a more predictable ‘model’ of disease onset and progression than are (selected) members of the general population.

Consequently, when comparing the origin and spread of tau and amyloid pathology (see Fig. 2) in this series of individuals with DS, it is clear that amyloid pathology occurred in the absence of tau pathology in 3 of 4 individuals aged between 13 and 35 years; neither were present in one 23-year old. Initially, this was present broadly to a similar extent in all regions of cerebral cortex, progressing with time into hippocampus, striatum and cerebellum. A pattern typical of AD [46] was reached by 50–55 years of age. These findings bear out our previous studies on more limited numbers of cases [28, 29] and are consistent with those of other workers where cases showing amyloid deposits in the absence of tau can be seen in individuals as young as 8 years of age [23, 24]. Early deposits of amyloid (Aβ) are seen as diffuse plaques and to be composed of the longer form Aβ_42(3)_, with the shorter form, Aβ_40_, only appearing later in (more advanced) cored plaques [20]. It has been suggested that deposition of amyloid in very young individuals with DS may have occurred in relationship to an institutionalized residential background [23]. In these present study, a mix of institutionalised and community based individuals were studied though no clear difference in pattern of amyloid deposition was seen between such individuals where this information was definitely available.

Interestingly, in the present study, amyloid deposition in blood vessel walls (CAA) did not commence before 42 years of age, some 30 years after initial deposition of Aβ as plaques. Similar findings have recently been reported by Head et al. [15]. It has been postulated that the strong association between age, CAA and AD pathology in the general population is driven, at least partially, by an impaired efficiency of cerebral vessels in later life in expelling extracellular fluid containing soluble forms of Aβ as a consequence of atherosclerosis/arteriosclerosis within such vessels [48]. However, individuals with DS appear to be protected against hypertension [1] and show less cerebrovascular pathology typically associated with cardiovascular risk factors, including atherosclerotic lesions and arteriolosclerosis [15, 27]. Paradoxically, this ought to result in a better preservation of perivascular drainage channels in DS, and consequently less severe CAA compared to aged persons in the general population with AD. Potential inefficiencies in perivascular drainage might only appertain to those few very elderly individuals with DS who might survive to an age bordering on those in the general population. However, Aβ can also be cleared from the brain through several other routes, involving endocytosis by microglia and astrocytes, or enzymatic degradation. It is therefore possible that failures in these latter pathways in younger persons with DS might foster an inability to expel Aβ and result in CAA.

As previously reported [18, 43, 44], *APOE* alleles and genotypes were similar to those in the general population, and in contrast to AD, there was no elevation of *APOE* ε4 allele (or reduction in ε2 allele) frequency. Nonetheless, again as reported previously [18, 41, 43, 51], age at death was earlier in ε4 allele bearers than in those without ε4 allele and was later in ε2 allele bearers than in those without ε2 allele suggesting that possession of *APOE* ε4 allele may hasten the onset of dementia and mortality by promoting an earlier formation, or a more rapid accumulation, of plaques and tangles. Although possession of *APOE* ε4 allele, and particularly homozygosity for this, has been reported to favour CAA at capillary level in persons with AD [37, 45, 47] we found no association in DS between *APOE* ε4 allele and type 3 CAA [2] (or type 2 CAA as in [47]).

On the other hand, tau pathology did not commence anywhere in the brain until after 35 years of age, though its onset in most cases appeared synchronous within both entorhinal/transentorhinal areas and subcortical nuclei (LC and DRN), with hippocampus and neocortical regions only becoming involved after 45–50 years of age. However, in two instances (cases #14 and 20) there was scant neuritic/neurofibrillary pathology in LC without any involvement of other cortical or subcortical structures. Such cases might be classed as ‘prodromal stages ‘a’ or ‘b’ [6, 8]. Nonetheless, thereafter pattern of spread of tau pathology in DS is similar to that seen in typical AD [4, 7]. Hence, observations in DS strongly validate present stageing protocols for tau and amyloid pathology, and provide the clearest ‘model’ yet for investigation the origins and progress of pathological changes typical of AD.

While most cases studied fitted in well with this age-increase pattern of spread of amyloid and tau pathology, though there were 2 notable exceptions, cases #20 and #39, which did not conform to pathological expectations given their chronological age. In case #20, aged 50 years, virtually no amyloid, and no tau pathology, was present, while in case #39 there was abundant amyloid pathology, though tau pathology was absent. It is possible that these individuals bore a karyotype other than full trisomy 21 since it is known that cases bearing only partial trisomy excluding the *APP* locus, but covering the obligate DS region, show little or no Alzheimer type pathology, even at advanced age [12, 40]. Unfortunately, a karyotype was not available for these 2 cases.

The low prevalence (9% cases) of TDP-43 pathological changes in hippocampus and fusiform gyrus is consistent with previous studies and parallels that seen in cases of sporadic and inherited early onset AD [11, 26]. This type of TDP-43 proteinopathy is much more common in late onset cases of AD [11, 34] at a time beyond the lifespan of people with DS. The higher prevalence of TDP-43 pathology in late onset AD compared to early onset AD and DS may be triggered as part of an age-associated process operating in conjunction with AD at a later time of life. Nonetheless, it is also possible that the presence of AD pathology per se initiates and promotes TDP-43 pathology. However, if this were so then a higher prevalence of TDP-43 pathology might be expected in DS than that which is seen given the severity of AD pathology occurring in older persons with DS.

A previous study has reported that α-synuclein immunopositive Lewy bodies and Lewy neurites are common in older individuals with DS, especially within the amygdaloid nucleus where as many as half of cases can show such changes [25]. In the present report, Lewy bodies and Lewy neurites were present in SN or LC or both in only 5 cases (9%) and were generally few or moderate in number. In these cases, no clear loss of nigral neurones had occurred. On the other hand, Lewy bodies and Lewy neurites were much more common in the entorhinal cortex and, to a lesser extent, temporal cortex, but again occurred only in 6 (11%) cases (which included the 5 cases where SN or LC was affected). Unfortunately, the amygdaloid nucleus was not available for investigation in the present study, though given the absence of Lewy body pathology in entorhinal cortex in most cases it is unlikely that these negative cases would have shown (at least florid) changes in the amygdala. The reason for the apparent lower frequency of Lewy body pathology in the present series of cases compared to that reported by Lippa and colleagues [25] is not clear given that both series examined individuals of a similar age range and employed α-synuclein immunostaining, albeit with different antibodies, though may be explained by the lack of investigation of the amygdala in our own study.

We did obtain some evidence (from cases #14 and 20) supporting the view [6, 11] that tau pathology in subcortical nuclei might predate that within entorhinal/transentorhinal areas, and that there might be stages in the tau pathological process before these latter cerebral cortical regions become affected. On the other hand, we observed that in 3 of the youngest individuals amyloid pathology preceded that of tau, and conversely that tau pathology was never seen in the absence of Aβ deposition – findings again consistent with the amyloid cascade hypothesis of AD [14]. Such findings accord with the view that pathological changes in tau might in some way be induced by damage to nerve terminals at the sites of amyloid plaque formation, with retrograde responses in the perikaryon leading to NFT formation. In the case of LC and DRN neurons this would occur at plaque sites within the cerebral cortex (and particularly the temporal cortex), since it is known that these nerve cell types project diffusely, but topographically, all cortical regions. Indeed, in DS and AD nerve cells of LC which project to cortical regions rich in plaques are lost whereas those projecting to relatively plaque free regions such as cerebellum, and plaque free spinal cord, are preserved [32, 33]. In entorhinal/transentorhinal areas induction of damage would take place at plaque sites within the molecular layer of the hippocampus where such neurons project via the perforant pathway [17]. The observations that tau pathology only became prevalent in SN after the age of 50 years – some 10–15 years after LC and DRN – would accord with the later deposition (relative to the cerebral cortex) of Aβ within the caudate nucleus and putamen, regions where these neurons make their principal connections.

On the other hand, it is possible that tau aggregation precedes and induces amyloid deposition in some way. Indeed, previous work based on cognitively normal young individuals suggests that areas such as LC might be the initial site of tau pathology in AD [6, 8]. However, the absence of amyloid deposits in the brains of such normal individuals implies a pathological process at work other than one that leads to AD, unless it is accepted that tau pathology in some way induces amyloid pathology at a later stage in persons other than those with DS. It is also possible that the tau pathology seen in these very young individuals is indicative of primary age related tauopathy (PART), where tau pathology occurs, especially in entorhinal/transentorhinal regions, in the relative or complete absence of amyloid deposition [10]. However, PART is generally associated with aged individuals, and an onset of this tau pathology in youth with maintenance through into old age seems unlikely. Tau pathological changes in LC and entorhinal cortex, in the absence of significant amyloid deposits, can also be seen in many patients with other neurodegenerative disorders such as Amyotrophic Lateral Sclerosis [3] or Huntington’s disease [21]. Furthermore, it is known that a reversible PHF-like pattern of tau pathology can be induced under certain physiological conditions such as hypothermia and anaesthesia [39]. Therefore, it remains possible that neurons of LC (as well as others such as those in DRN and nucleus of Meynert) are exquisitely vulnerable to a range of physiological or neurotoxic changes that induce tau pathology, and that these putative early changes in tau in LC in healthy young people in the absence of amyloid deposits [6, 8] might simply be serendipitous or even transient, never progressing into a pathological process culminating in AD.

Finally, it is possible that the initiation of tau and amyloid pathologies occur independently but once started each process might act synergistically to promote the progression of the other. It might even be the case that onset of tau pathology in persons with DS represents early onset of aging processes since it is well known that such individuals display other aspects of ‘precocious aging’ involving skin, hair or cardiac changes at an earlier time of life compared to people within the general population.

## Conclusion

In conclusion, a study of the brains of individuals with DS at different ages reinforces the concepts underlying the amyloid cascade hypothesis of AD and supports present stageing protocols for the onset and spread of both amyloid and tau pathology.
